# Non-Invasive Evaluation of Heart Function with Four-Dimensional Echocardiography

**DOI:** 10.1371/journal.pone.0154996

**Published:** 2016-05-04

**Authors:** Ran Chen, Meihua Zhu, David J. Sahn, Muhammad Ashraf

**Affiliations:** 1 Department of Diagnostic Ultrasound and Echocardiography, Sir Run Run Shaw Hospital, Zhejiang University College of Medicine, Hangzhou, China; 2 Division of Pediatric Cardiology, Oregon Health & Science University, Portland, OR, United States of America; Northwestern University, UNITED STATES

## Abstract

**Background:**

The aim of this study is to assess the accuracy and feasibility of left ventricular systolic function determined by four-dimensional echocardiography (4DE).

**Methods:**

Latex balloons were sewn into the left ventricle (LV) of 20 freshly harvested pig hearts which were then passively driven by a pulsatile pump apparatus. Global longitudinal strain (GLS), global circumferential strain (GCS), global area strain (GAS) and left ventricular ejection fraction (LVEF) derived from 4DEand two-dimensional echocardiography (2DE)-derived LVEF were quantified at different stroke volumes (SV) 30–70 ml and correlated with sonomicrometry data.

**Results:**

In all comparisons, GLS, GCS, GAS, 2DE-LVEF, and 4DE-LVEF demonstrated strong correlations with sonomicrometry data (r = 0.77, r = 0.89, r = 0.79, r = 0.93, r = 0.96, all P <0.001). Bland-Altman analyses showed slight overestimations of echo-derived GLS, GCS, 2DE-LVEF and 3DE-LVEF over sonomicrometry values (bias = 2.88, bias = 3.99, bias = 3.37, bias = 2.78, respectively). Furthermore, there is better agreement between GCS, 4D LVEF and sonomicrometry values compared with GLS and 2D LVEF.

**Conclusion:**

Four-dimensional echocardiography accurately assesses LV function. GCS derived by 4DE is a potential alternative parameter to quantify LV systolic function.

## Introduction

Left ventricular (LV) systolic function is an important predictor of cardiac mortality and morbidity and often is used to select patients for surgical and pharmacological treatment [1,2]. LV ejection fraction (LVEF) remains the most commonly used conventional parameter to quantify global LV function in patients. Despite its widespread clinical use, assessment of LVEF is limited by problems associated with load dependence, reproducibility, LV geometric assumptions and dependence on operator expertise [3, 4]. Global LV systolic function is the result of a complex myocardial fiber contraction. Myocardial fibers consist of right-handed helical geometry in the subendocardial layer of the myocardial wall which gradually changes into left-handed geometry in the subepicardial layer [5, 6]. Its contraction determines changes of LV size and shape, which are the result of longitudinal shortening, circumferential rotation, and radial thickening of the myocardium. In many different cardiac diseases, LVEF is regarded as a global index of LV function, ignoring the relative role of the different components of myocardial motion, which may be influenced to a different extent in early stage of diseases with normal LVEF [7].

Strain is a useful index for determining LV regional and global systolic function in research and clinical settings. Two-dimensional (2D) speckle-tracking echocardiography (STE), a novel technique to quantify the complex cardiac motion based on frame-to-frame tracking of acoustic speckles in grayscale 2D images, has been shown to be superior to LVEF in the quantification of LV segmental wall motion and LV global function. However, 2D STE is limited by its geometric assumptions, while myocardial mechanics of LV function are complex, involving multidirectional axes of motion. 2D STE is unable to quantify one of the three components of the local displacement vectors. The newly developed 3D speckle tracking program is the most direct and simple way of acquiring 4D cardiac data. 4DE overcomes the 2D limitations because it allows the acquisition of a full 3D volumetric data set with simultaneous evaluation of multidirectional wall motion. Yu et al [8] demonstrated that 3D strain (3DS), which is best described as the “principle tangential strain”, may display the resulting direction’s muscle fiber contractions, which are aligned either parallel to or tangential to the myocardial surface.

Previous studies demonstrated the improved accuracy and reproducibility of LV functional quantification using real time three-dimensional echocardiography (RT3DE) or 4DE compared to those generated via 2D acquisition [9–11]. RT3DE utilizes a matrix array of more than 3000 elements to record several full-volume images of the LV over the course of a cardiac cycle and relies on 3D echocardiographic datasets, tracking the motion of acoustic speckles within a scanned volume, irrespective of direction [12, 13]. In addition, LVEF obtained by speckle tracking imaging that allows evaluation of 3D LV anatomy will overcome the limitation of geometric assumptions associated with 2D LVEF. However, there is no study to demonstrate the accuracy of the 4DE on strain determination and LVEF calculation against the reference sonomicrometry data [14, 15].

The Toshiba Aplio Artida ultrasound system with a matrix array PST-25SX 2.5 MHz transducer is capable of acquiring 4D volumes, and offline analysis software allows analysis of 4DE datasets to provide quantification of strain and LVEF. The aim of our study was to validate 4D echocardiography as an alternative quantifier of LV function by comparing the accuracy of three different components of global strain: global longitudinal strain (GLS), global circumferential strain (GCS), global area strain (GAS) and LVEF with sonomicrometry values as a gold standard. Area strain is a relatively new parameter, which integrates both longitudinal and circumferential deformation, and is suggested as a sensitive and reproducible parameter to detect early and subtle LV systolic dysfunction, showing greater feasibility than other conventional strain parameters [16].

## Materials and Methods

A closed-circuit pulsatile harvested pig heart model was designed for this study. Pig hearts were purchased from Carlton Farms (Carlton, Oregon), which processes pork for commercial sale and is licensed to provide organs for in vitro research. OHSU does not require IACUC approval for in vitro projects.

A latex balloon was sewn into the mitral valve annulus of the LV in twenty freshly harvested pig hearts with atria and major vessels removed. The aortic outlet was sutured closed to prevent the inflated balloon from exiting the LV. The base of the heart was mounted on a plastic ring to minimize global movement during the simulated cardiac cycle while remaining outside of the imaging window to reduce artifacts. A calibrated pulsatile pump (Harvard Apparatus, South Natick, MA, USA) was connected using two plastic tubes to a Y-shape connector, which was connected to a latex balloon. The balloon was filled with water until the passive resistance of the LV cardiac muscle was sufficient to oppose the gravitation force of the water before all of the air bubbles were removed. This apparatus and a transducer were fixed inside a water tank. The distance between LV apex and the transducer was adjusted to obtain the best image quality. The pump defined stroke volume (SV) ranged from 30 to 70 ml.

Four sonomicrometry crystals were sewn into the myocardium through the epicardium. Crystals 1 and 2 were placed in the base of LV anterior wall and in the apex of LV, respectively. Crystals 1–2 were longitudinally oriented along the long axis of the LV to determine longitudinal strain (LS) and long axis dimensions. Crystals 3 and 4 were sewed at the middle of LV interventricular septum and lateral wall. Crystals 3–4 were perpendicular to the line of crystals 1–2 to calculate circumferential strain (CS) and short axis dimensions.

Signals from the sonomicrometry crystals were acquired using SonoSoft software (SonoMetrics, London, Ontario, Canada) as a reference standard for EF and echo-derived stain. The LV volumes were calculated as a modified ellipsoid model with the use of the following formula V_LV_ = (π/6) ·D_long_·D_short_·D_short_, (V_LV_; volume of LV, D_long_; long-axis LV dimension, D_short_; short-axis of LV dimension). Ejection Fraction (EF) was calculated from the difference between the maximum LV volume (EDV) and the minimum LV volume (ESV), using formula: [(EDV—ESV) / EDV] x 100 = EF (%).

Full 4D volumes were acquired with Toshiba Aplio Artida ultrasound system (Toshiba Medical Systems, Tochigi, Japan) with a matrix array PST-25SX 2.5 MHz transducer. Gain was determined for the best endocardial border definition. Depth and scanning angle were adjusted to include the entire LV and maximize temporal resolution. In this study, SV ranged from 30 to 70 mL at an increment of 10 mL with a constant end-systolic volume maintained.

**Fig 1:**
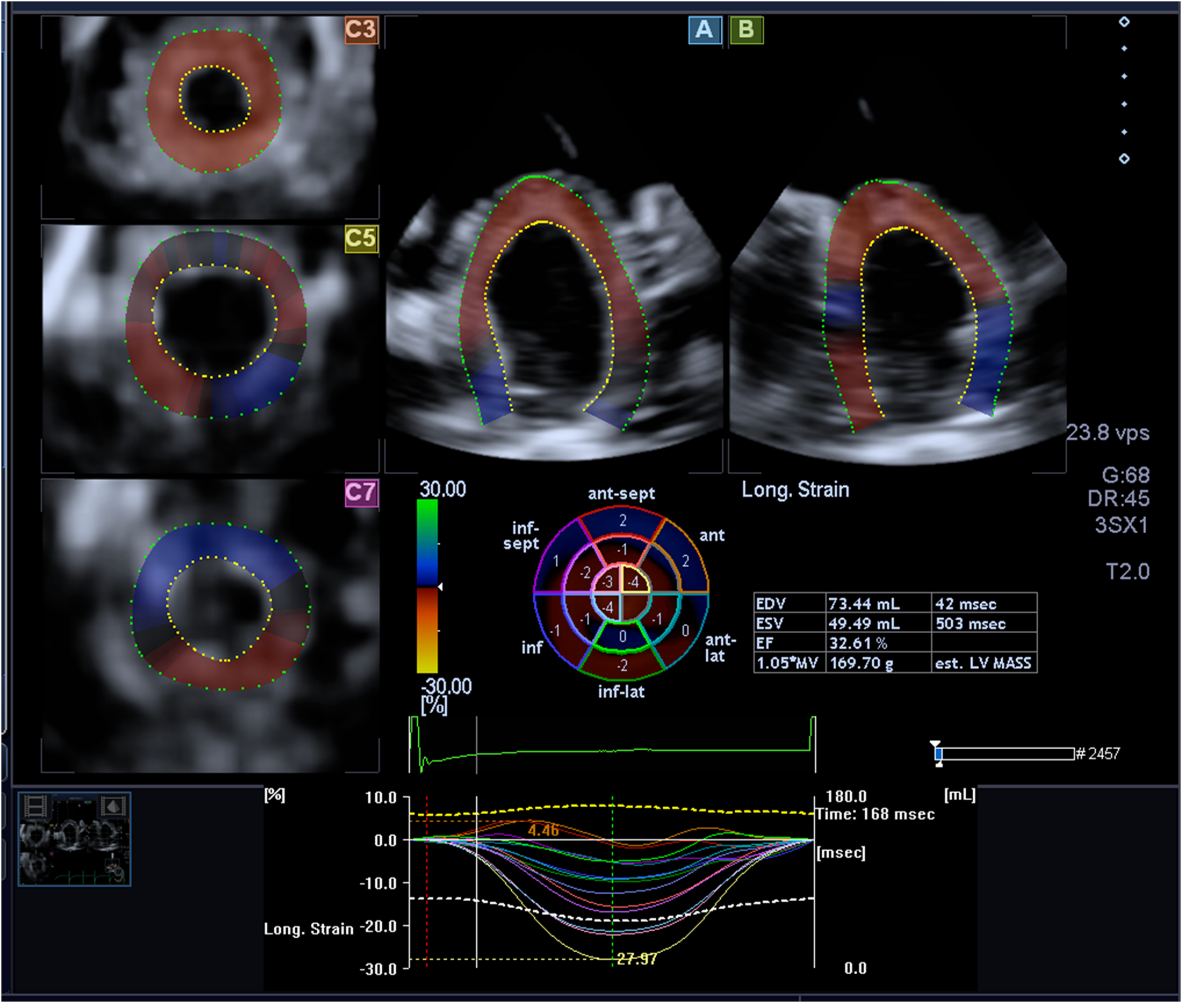
Strain Values derived by three-dimensional speckle tracking program: 3D datasets were displayed in 2 cross-sectional planes. Panel A and B showed longitudinal apical four-chamber and two-chamber views displayed on the right side. 3 short-axis views were displayed on the left side, which were redefined by adjusting the lines in both apical longitudinal views at different short-axis levels (apex, middle, base) to get clear cardiac borders. The region of interest was defined by points at the right and left mitral annulus as well as the apex at end-diastole in apical four-chamber and two-chamber views; the program automatically generated endocardial and epicardial border lines which were manually adjusted in all long-axis planes and short-axis planes. 3D strain parameters could be directly acquired in an exported Excel spreadsheet.

Full 4D images were analyzed by a three-dimensional speckle tracking program in which the endocardial and epicardial border can be automatically generated and then manually adjusted to refine the tracing at the following five views: (1) the apical four-chamber view; (2) the apical two chamber view and the three short-axis views; (3) the apex of the LV; (4) the midlevel of the LV; and (5) the basal level of the LV. Thus, the software was able to perform all deformation parameters including global longitudinal strain (GLS), global area strain (GAS), and global CS (GCS) throughout the entire cardiac cycle. Meanwhile, left ventricular ejection fractions (LVEF) were observed automatically (Fig 1).

### Reproducibility

To test intraobserver reproducibility, a single observer analyzed the data twice on occasions separated by an interval of 1 month for the measurement of GLS, GCS, GAS and LVEF from 4DE and sonomicrometry in 10 subjects. To test interobserver variability, a second observer analyzed the data in 10 subjects without knowledge of the first observer’s measurements.

### Statistical Analysis

All statistical analyses were performed using SPSS version 16.0software (SPSS, Inc., Chicago, IL, USA). Linear regression analysis was applied between strain values derived by 3D speckle tracking program and different SV. Pearson’s correlation analysis was performed between sonomicrometry data and echo-strain values, and also applied between sonomicrometry data and LVEF derived by 2D and 4D echocardiography. The agreements of sonomicrometry data against strain value and LVEF derived by 2D and 4D echocardiography were assessed by Bland-Altman analysis. Intra-observer variability and inter-observer reliability was assessed using the intraclass correlation coefficient (ICC) along with absolute differences, expressed as the mean percentages error.

## Results

**Fig 2:**
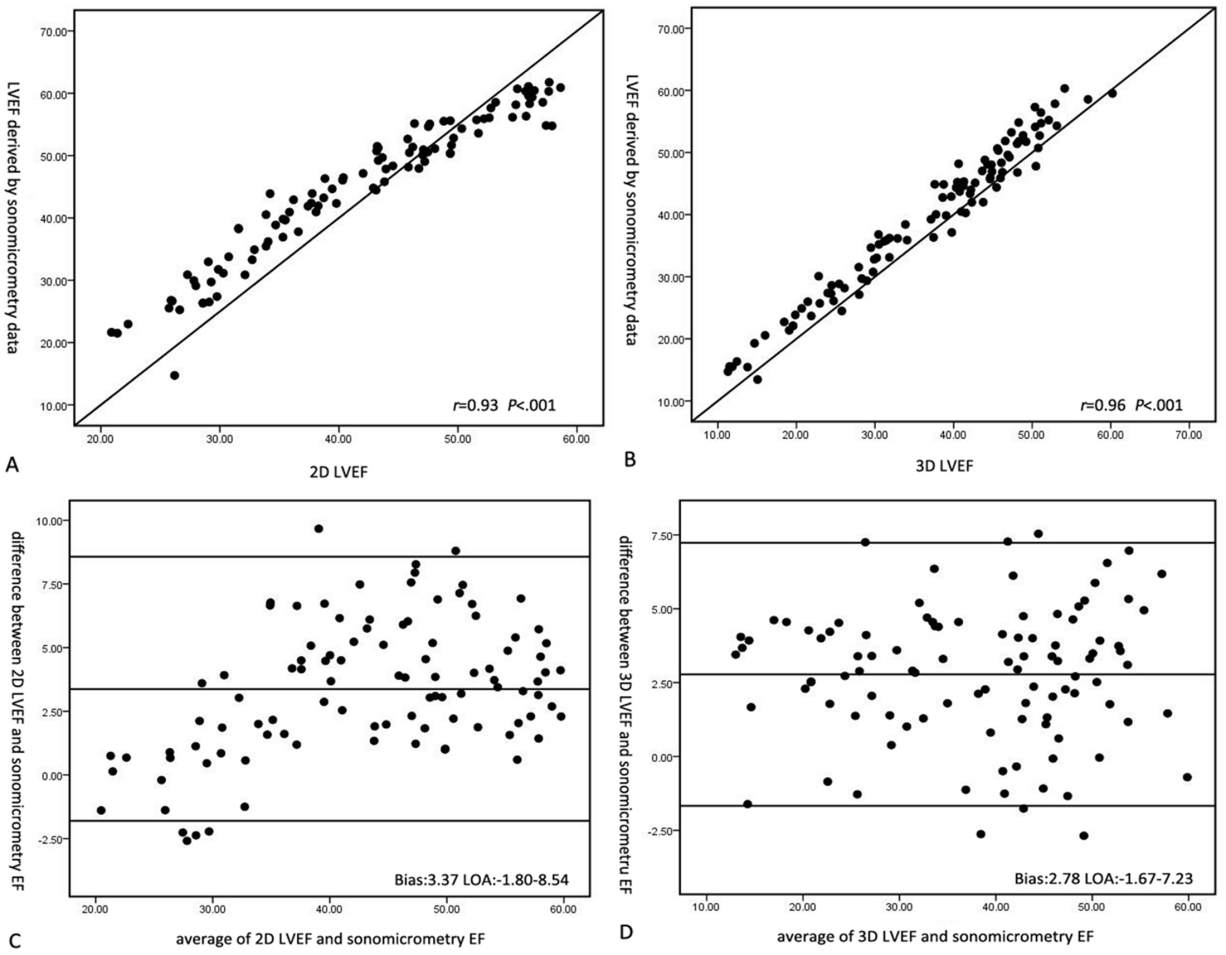
Linear correlation and Bland-Altman analyses of 2D LVEF, 4D LVEF and EF derived by sonomicrometry volume data. A. The correlation analysis of 2D LVEF and sonomicrometry values; B. The correlation analysis of 4D LVEF and sonomicrometry values; C. Bland-Altman analysis of 2D LVEF and sonomicrometry values; D. Bland-Altman analysis of 4D LVEF and sonomicrometry values.

Linear regression analysis and Bland-Altman analysis showed excellent correlations and strong agreements between 2D LVEF, 4D LVEF and EF derived by sonomicrometry data. Furthermore, the correlation coefficient between 4D LVEF and sonomicrometry demonstrated better linear correlation than that of 2D LVEF and sonomicrometry data (2D LVEF vs sonomicrometry data: r = 0.93, P< 0.001; 4D LVEF vs sonomicrometry data: r = 0.96, P< 0.001). Bland-Altman analysis also displayed higher overestimation of 2D LVEF (3.37) than 3D LVEF (2.78) when compared with sonomicrometry (Fig 2).

**Fig 3:**
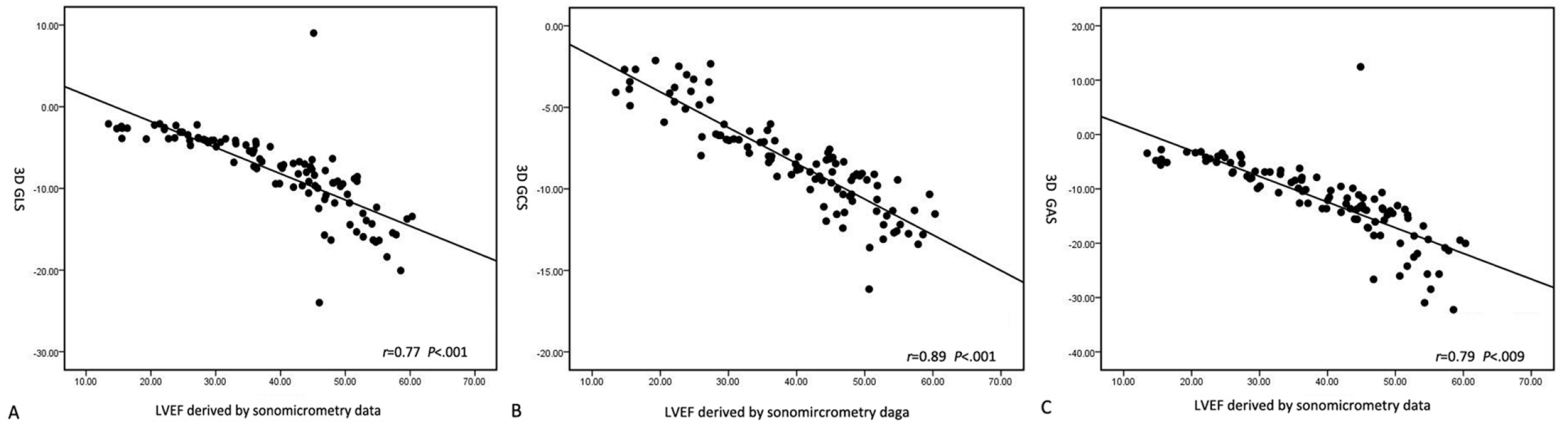
The correlation analysis of strain values derived by 3D speckle tracking program and EF obtained by sonomicrometry data. A. The correlation of 3D GLS and sonomicrometry data; B. The correlation of 3D GCS and sonomicrometry data; C. The correlation of 3D GAS and sonomicrometry data.

**Fig 4:**
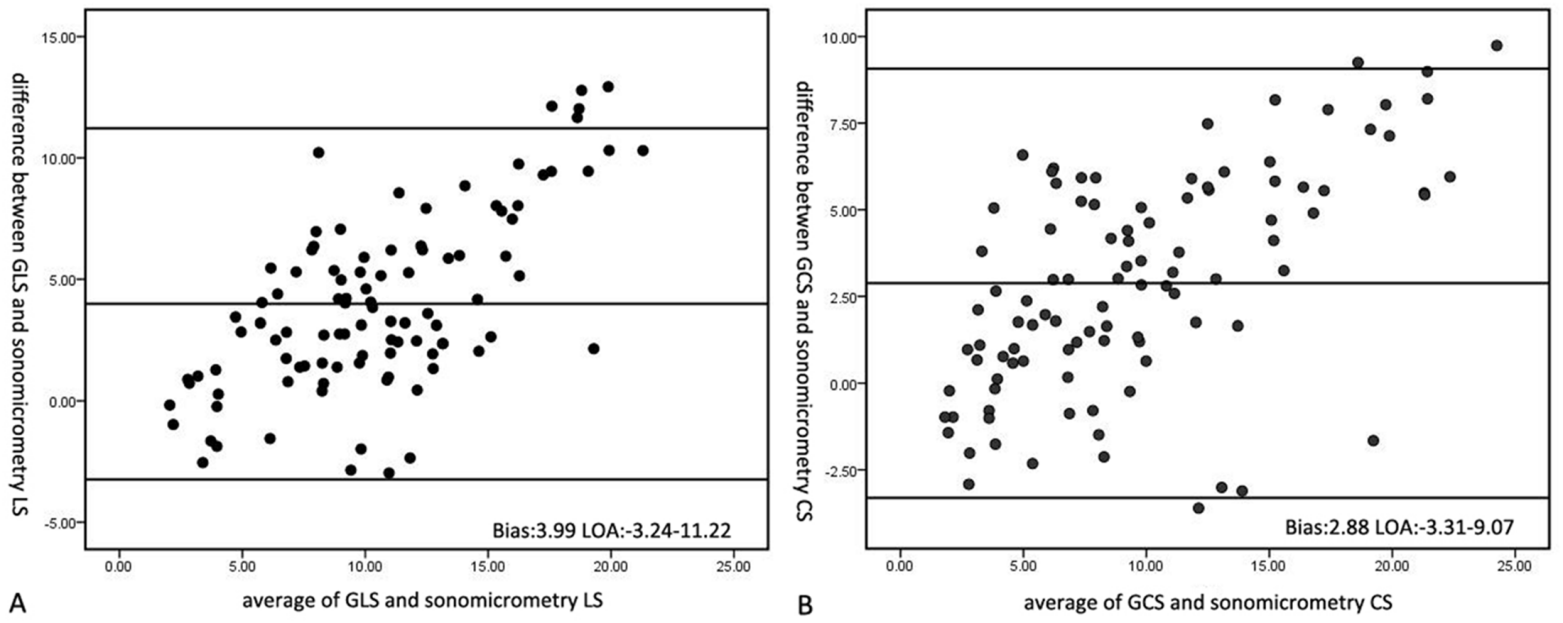
Bland-Altman analysis of strain values by 3D speckle tracking program and strain value derived by sonomicrometry data. A. Bland-Altman analysis of 3D GLS and strain value derived by sonomicrometry data; B. Bland-Altman analysis of 3D GCS and strain value derived by sonomicrometry data.

Strain values from 4DE and sonomicrometry data also demonstrated good correlations. The strongest correlate for EF obtained by sonomicrometry was with 3D GCS (r = 0.89, P> 0.001). The correlation result for 3D GAS was r = 0.79, P< 0.001. The weakest correlation for EF was with 3D GLS (r = 0.77, P< 0.001)(Fig 3). Strong agreement between 3D strain values and sonomicrometry strain data was demonstrated using Bland-Altman analysis. It showed slight overestimation of GLS and GCS against sonomicrometry data. However, there was better agreement between GCS and sonomicrometry than between GLS and sonomicrometry (GCS: bias = 2.88; GLS: bias = 3.99, respectively)(Fig 4).

**Fig 5:**
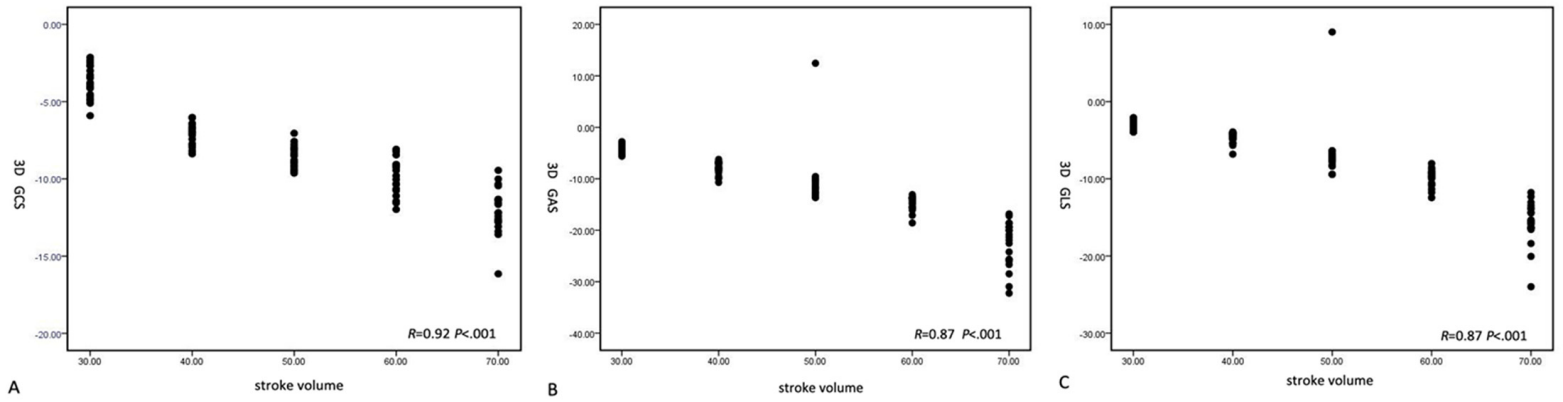
Line regression analysis of strain values obtained by 3D speckle tracking program and SV. A. Line regression analysis of 3D GCS and SV; B. Line regression analysis of 3D GAS and SV; C. Line regression analysis of 3D GLS and SV.

**Fig 6:**
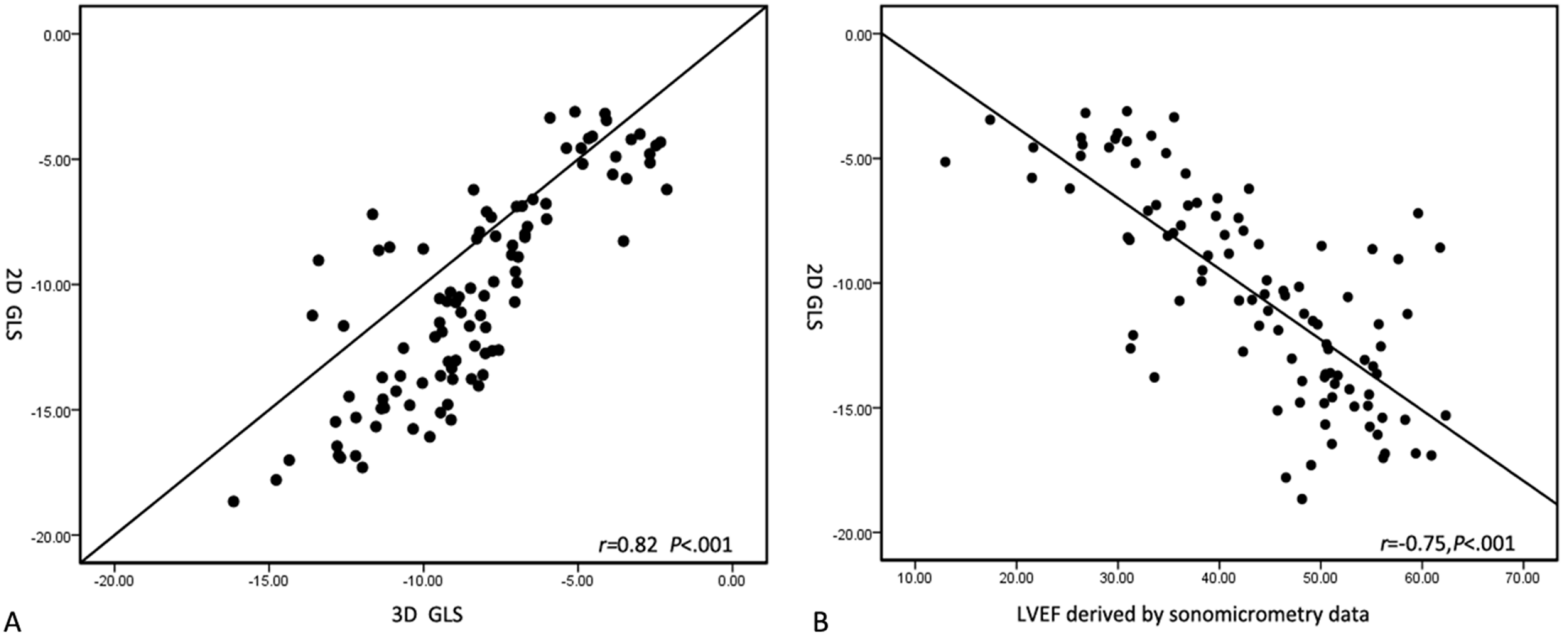
Correlation analysis of strain values by 2D, 4D speckle tracking program and sonomicrometry data. A. Correlation analysis of 2D GLS and strain value derived by 4DE; B. Correlation analysis of 2D GLS and sonomicrometry data.

3D GLS, GCS and GAS derived by 3D speckle tracking program continuously increased with the increment of SV. Linear regression analysis indicated the highest regression coefficient appeared between 3D GCS and SV (GCS: r = 0.92, P< 0.001; GLS: r = 0.87, P< 0.001; GAS: r = 0.87, P< 0.001) (Fig 5). GLS was used to analyze the correlation between 2D strain, 4D strain and sonomicrometry data. There were still good correlation between 2D GLS and 4D GLS (r = 0.82, P< .001); and between 2D GLS and sonomicrometry data (r = 0.75, P < .001) (Fig 6).

### Interobserver and Intraobserver Reproducibility

Good reproducibility was shown for both intraobserver and interobserver reliability (Table 1), with ICCs ranging from 0.80 to 0.92. The mean absolute percentage error of all the measurements did not exceed 0.1.

**Table 1:** Reproducibility of interobsever and intraobserver for strain value and EF from 4DE and sonomicrometry.

	Interobserver		Intraobserver	
	ICC	MAPE	ICC	MAPE
GLS	0.81	0.06	0.82	0.03
GCS	0.83	0.04	0.80	0.05
CAS	0.82	0.05	0.92	0.04
2D LVEF	0.86	0.06	0.88	0.09
4D LVEF	0.80	0.05	0.84	0.03
Sono EF	0.91	0.07	0.87	0.07
Sono LS	0.83	0.08	0.82	0.06
Sono CS	0.82	0.08	0.81	0.03

GLS = global longitudinal strain; GCS = global circumferential strain; GAS = global area strain ICC = intraclass correlation coefficient; 2D LVEF = left ventricular ejection fraction derived by two dimensional echocardiography; 4D LVEF = four dimensional echocardiography; Sono EF = ejection fraction from sonomicrometry; Sono LS = longitudinal strain from sonomicrometry; Sono CS = circumferential strain from sonomicrometry; MAPE = mean absolute percentage error.

## Discussion

Accurate quantification of LV systolic function has important prognostic implications and is helpful to determine treatment decisions for a variety of therapies. 2D LVEF is the most commonly used echocardiographic parameter to evaluate the LV function in clinical practice. However, measurement of LVEF is limited by geometric structure, image quality, load dependence and poor reproducibility [17]. Therefore, it is necessary to develop a more sensitive and accurate technique to quantify LV systolic function.

2D STE has been validated by cardiac MRI and 3D echocardiography as an effective method to assess LV function [18]. Many previous studies have shown that 2D STE provides more accurate prognostic implications than traditional 2D LVEF in assessment of a variety of clinical heart diseases, such as heart failure [19–21], valvular heart disease [22, 23], ischemic heart disease [24–26]. However, 2D STE has the potential limitation of out-of-plane motion tracking of speckles, which can lead to increased noise and reduced accuracy [27, 28].

4D echocardiographic techniques including real-time 3D speckle tracking program and 3D echocardiography allow volumetric analysis and simultaneous measurement of multidirectional components of strain in a single data set. The acquisition of the entire LV within a single data set allows global assessment of LV longitudinal, circumferential and radial functions across all myocardial segments. Zhu et al [10] demonstrated that real-time 3D wall motion tracking has the ability to accurately determine regional myocardial deformation and detect myocardial infarction in 20 freshly harvested pig hearts.

In previously published literature, there is strong correlation between LVEF and volumetric assessment obtained by 3D STE and magnetic resonance imaging (MRI) [29]. Jenkins et al [13] also showed that real-time 3DE is a feasible approach to improve accuracy of LV volume, EF, and mass measurements in the assessment of LV. Sonomicrometry is a technique of measuring the distance between piezoelectric crystals based on the speed of acoustic signals through the medium they are embedded in. An electrical signal sent to either crystal will be transformed in to sound, which passes through the medium, eventually reaching the other crystal, which converts the sound into electricity, detected by a receiver. From the time taken for sound to move between the crystals and the speed of sound in the medium, the distance between them was calculated. Sonomicrometry is a well-established technique to study pressure-volume loops in animals. They are most commonly implanted within cardiac muscle tissue to track changes in distance and to estimate left ventricular volume according to a geometric algorithm. Several studies have documented accuracy of this method of studying LV volumes [10,11,30–33]. So sonomicrometry often was used as the gold standard to quantify regional myocardial function in previous studies [34]. This study is the first one to evaluate the LVEF using 4DE comparison with sonomicrometry. This study demonstrated stronger correlations and higher agreement between 3D LVEF and EF obtained by sonomicrometry than that obtained by 2D LVEF and EF derived by sonomicrometry EF. We believe that 4DE overcomes the geometrical limitations and insufficient image quality for endocardial tracking of 2DE in evaluation of LVEF. The similar study was presented in our previous study. Zhu et al [11] showed the feasibility and accuracy of nongated four-dimensional echocardiography for determining LV SV and mass in a fetal heart-size LV model compared with sonomicrometry.

Our results showed GLS, GCS, and GAS from 4DE increased by the increment of SV (30ml to 70ml). In other words, GLS, GCS, and GAS could increase by the increment of LVEF. And they were all correlated well with LVEF obtained by the gold standard of sonomicrometry. Especially, there was a stronger correlation between GCS and LVEF than GLS and GCS. Therefore, GCS is an alternative parameter to quantify LV systolic function. It is postulated that circumferential fibers play a more important role in LV myocardial function than that of longitudinal fibers because circumferential fibers, which are over longitudinal fibers within the myocardial wall, exist in greater proportion in the basal segments compared with the apex. This result was similar as a study by Luis et al [17], which demonstrated 3D GCS was a better marker of LV function than 3D GAS and GLS and existed higher prediction of LV dysfunction with 92% sensitivity and 90% specificity by comparison 3D strain values with LVEF obtained by two-dimensional and real-time three-dimensional echocardiography, however, not compared with LVEF from sonomicrometry data. In contrast to previously published literature, our study is the first to demonstrate a strong correlation between strain values from 4DE and sonomicrometry. We extrapolated the value of GCS from 4DE could be a prognostic parameter beyond GLS and GAS in future studies.

The results in this study also showed strong agreement between strain values and sonomicrometry strain data and there was better agreement between GCS and sonomicrometry than between GLS and sonomicrometry. Seo et al [35] assessed regional left ventricular myocardial function by real time three-dimensional speckle tracking imaging against data obtained by sonomicrometry at baseline and during pharmacological stress test and acute myocardial ischemia induced by coronary artery occlusion. The results demonstrated there was strong correlation and agreement between real-time 3D tracking images derived and sonomicrometry derived CS and LS in all measurements.

In our results, Bland-Altman analyses demonstrated a slight overestimation between 3D strain values and sonomicrometry strain data, which is similar to our previous studies [10,11]. The reason is that strain at the epicardium is relatively lower than at other myocardium layers [36, 37]. In our study, sonomicrometry crystals were secured into the myocardium through the epicardium, which may have resulted in underestimated sonomicrometry strain values. In the course of the speckle tracking of the entire porcine heart myocardial wall to calculate strain, GLS and GCS should be greater than the strain at the epicardium; thus, the overestimation of strain was unavoidable.

### Limitations

Although the harvested pig heart model could model the unique imaging properties of cardiac tissue at a similar size to the human heart, the model itself is not a perfect replica in terms of chamber shape, contractility, and cardiac mechanics. The limitation of 4DE is related to the relative low temporal resolution, which is still inferior to that of 2DE. Technological advances increasing the temporal resolution of 4DE would improve the accuracy of LV quantification.

A recent study demonstrated the strain parameters follow a shape function correlation to EF, not necessarily linear [38], which was obtained by the mathematic model analysis based on the data from thousands of samples. The purpose of our data was to investigate the correlations between GLS, GCS, GAS and EF and the number of data is not adequate to build a mathematic model. So a lot of data and a more stringent model need to be discussed in our later study. In addition, the accuracy of sonomicrometry for evaluation of strain and volume would be influenced by the sewing position and method. The LV volume was calculated from the distance of sonomicrometry crystals. The orientation of the crystals may affect the volume calculation. Furthermore, the crystals were sewed into myocardium from the epicardium to avoid balloon rupture. So strain values from the myocardium are different from endocardial strain.

## Conclusions

4DE is a feasible modality that has the ability to accurately assess the LVEF and multidirectional components of strain of the LV.GCS derived by 4DE is a potential alternative marker to quantify LV systolic function.
